# *k*-nonical space: sketching with reverse complements

**DOI:** 10.1093/bioinformatics/btae629

**Published:** 2024-10-21

**Authors:** Guillaume Marçais, C S Elder, Carl Kingsford

**Affiliations:** Ray and Stephanie Lane Computational Biology Department, Carnegie Mellon University, Pittsburgh, PA 15213, United States; Ray and Stephanie Lane Computational Biology Department, Carnegie Mellon University, Pittsburgh, PA 15213, United States; Ray and Stephanie Lane Computational Biology Department, Carnegie Mellon University, Pittsburgh, PA 15213, United States

## Abstract

**Motivation:**

Sequences equivalent to their reverse complements (i.e. double-stranded DNA) have no analogue in text analysis and non-biological string algorithms. Despite this striking difference, algorithms designed for computational biology (e.g. sketching algorithms) are designed and tested in the same way as classical string algorithms. Then, as a post-processing step, these algorithms are adapted to work with genomic sequences by folding a *k*-mer and its reverse complement into a single sequence: The canonical representation (*k*-nonical space).

**Results:**

The effect of using the canonical representation with sketching methods is understudied and not understood. As a first step, we use context-free sketching methods to illustrate the potentially detrimental effects of using canonical *k*-mers with string algorithms not designed to accommodate for them. In particular, we show that large stretches of the genome (“sketching deserts”) are undersampled or entirely skipped by context-free sketching methods, effectively making these genomic regions invisible to subsequent algorithms using these sketches. We provide empirical data showing these effects and develop a theoretical framework explaining the appearance of sketching deserts. Finally, we propose two schemes to accommodate for these effects: (i) a new procedure that adapts existing sketching methods to *k*-nonical space and (ii) an optimization procedure to directly design new sketching methods for *k*-nonical space.

**Availability and implementation:**

The code used in this analysis is available under a permissive license at https://github.com/Kingsford-Group/mdsscope.

## 1 Introduction

Genomics sequence analysis shares many similarities with text analysis since they are both concerned with efficiently storing and searching long strings. Consequently, many algorithms are common between the two fields. The double-stranded nature of DNA brings a unique twist to genomics sequence analysis: A sequence and its reverse complement, in many cases, should be considered identical. For example, a sequencing read can represent either strand of a chromosome, and a read aligner attempts to find the best alignments of the read and its reverse complement against a genome. This concept has no equivalent in text analysis.

Sketching methods [e.g. minimizers (Roberts et al. 2004a,b), syncmers (Edgar 2021)] create a small representation of a sequence (a “sketch”) by selecting a subset of *k*-mers (substrings of fixed length *k*) from the original sequence. Because of their small size, sketches allow for efficient sequence similarity estimation that is much faster than comparing the original sequences. Thus, sketching is a common strategy to make bioinformatics algorithms more efficient (see Marçais et al. 2019 and Zheng et al. 2023 for reviews). To handle reverse complements, sketches usually work with “canonical *k*-mers,” i.e. the smallest (lexicographically) of *m* and the reverse complement ${\bar{m}}^{r}$. By using this canonical representation, denoted by $m^{c}$, a *k*-mer and its reverse complement are treated equally.

More precisely, for our purposes, a sketching method is a function φ that takes as input one or more *k*-mers (the context) and outputs a (possibly empty) set of the indices of the *k*-mers to select. A sketch of a sequence *S* is constructed by collecting all the *k*-mers selected by φ over all the contexts of *S*. Sketching methods are usually designed and evaluated as string algorithms working on standard strings. Then, to handle reverse complements, implementations of sketching methods do not use φ directly but rather work in “*k*-nonical space:” the selection function is composed with the canonical function, i.e. instead of querying φ(m) one queries $\varphi (m^{c})$. See e.g. the minimap (Li 2016, Li and Birol 2018) aligner using minimizers and the modified version using parameterized syncmers (Dutta et al. 2022), or genome assembly de Bruijn graphs built with universe minimizers (Ekim et al. 2021).

There exist many sketching functions, and their performance has been studied in diverse settings; however, the effect of using canonical *k*-mers has received little attention. This is surprising given the importance of reverse complements to computational biology. Moving to *k*-nonical space has been primarily viewed as an implementation detail that is not likely to have an impact on downstream applications. Unfortunately, as we show, moving to canonical space can have a significant impact, in particular for *context-free* sketching methods.

An important property of sketching methods is the *window guarantee*, i.e. the assurance that the distance between two selected *k*-mers in an input sequence *S* is not too large. There are nuances to what this guarantee entails (e.g. if it is probabilistic, if some subsequences are excluded, etc) that we discuss in Section 2.2. In essence, it means that *k*-mers are selected at approximately regular intervals from an input sequence and that no large regions of the genome are ignored. Subsequent algorithms that use sketching typically require the window guarantee to prove their correctness.

This article has two main contributions. First, we show in Section 3 that using a sketching method in canonical space may not preserve the original method’s window guarantee. The lack of window guarantee can create “sketching deserts,” i.e. regions of the genome where few or no *k*-mers are selected. Consequently, these regions are ignored or under-represented in subsequent analysis (e.g. missing alignments), which can create unexpected statistical biases.

As is seen in Section 3.3, this problem mainly affects “context-free” sketching methods (e.g. syncmers), while “context-aware” methods (e.g. minimizers) are by construction not subject to this issue (see Section 2 for definitions). Consequently, we focus on context-free methods and Section 3 gives theoretical reasons and empirical evidence for these sketching deserts when using context-free methods in *k*-nonical space.

The second contribution is two procedures to generate context-free methods that are robust (i.e. do not have new sketching deserts) in *k*-nonical space. The first is a greedy procedure that adapts an existing sketching method to use canonical *k*-mers in a way that does not create new sketching desert. The second method is an integer linear program (ILP) optimization to generate *de novo* sketching methods that by construction handle *m* and ${\bar{m}}^{r}$ identically. These methods are still computationally intensive and may not without further optimization scale to large values of *k*.

Using canonical *k*-mers is a common way to handle reverse complemented sequences. Furthermore, because of the desirable properties [e.g. better conservation (Edgar 2021)] of context-free sketching methods, there is a surge in theoretical interest (Edgar 2021, Dutta et al. 2022, Shaw and Yu 2022) and these methods are increasingly used in bioinformatics tools [e.g. Ekim et al. 2021; Rautiainen et al. 2023; Shaw and Yu 2023]. To avoid creating undesirable behaviors with these tools, it is therefore important to ensure that the sketching method used is immune to the effects of using canonical *k*-mers, e.g. by using context-aware methods or context-free methods designed to be robust.

More generally, algorithms handling sequencing data must be designed to work properly in the presence of sequences and their reverse complements, and not only as classical string algorithms.

## 2 Preliminaries

### 2.1 Notations

All sequences are strings over the alphabet Σ of size σ=|Σ|, and Σ={0,…,σ−1}. For the sake of simplicity, we assume that *σ* is even (*σ*  =  2 or *σ*  =  4 in our examples). Moreover, every letter of the alphabet has a complement: a,b∈Σ are complements of each other when a+b=σ−1. This is denoted as $a=\bar{b}$ or (equivalently) $b=\bar{a}$. The genomic case corresponds to the mapping A=0,C=1,G=2,T=3. A sequence is an element of $\Sigma^{*}$, and S[i:c] is the substring of *S* starting at offset *i* and of length *c*.

Given a *k*-mer $m=m_{1}\ldots m_{k}$, its reverse complement is the *k*-mer ${\bar{m}}^{r}=\bar{m_{k}}\ldots \bar{m_{1}}$. The set ${\bar{A}}^{r}$ contains the reverse complemented *k*-mers of set *A*. The *canonical representation* of *m* is $m^{c}=\min(m,{\bar{m}}^{r})$, i.e. the lexicographically smallest of *m* and ${\bar{m}}^{r}$. The sets C and ${\bar{C}}^{r}$ denote canonical *k*-mers and their reverse complements, respectively. These sets cover the *k*-mers (i.e. $\Sigma^{k}=C\cup {\bar{C}}^{r}$) but may not be disjoint. Specifically, when *k* is even, the intersection $C\cap {\bar{C}}^{r}$ is the set of self-reverse complement *k*-mers—e.g. *k*-mers such as 0011 in the binary alphabet.

In general, a sketching method is a function

$$\varphi :\overset{c\;\text{input}\;k-\text{mers}}{\overset{︷}{\Sigma^{k}\times \cdots \times \Sigma^{k}}}\to P([1,c]),$$

where P is the power set. That is, the sketching function takes *c k*-mers as input (the context) and returns a possibly empty list of *k*-mer positions to select from this context. The context of length *c* of *S* at position *i* is the list of the *c* consecutive *k*-mers starting at position *i*: C(S,c,i)=[S[i:k],…,S[i+c−1:k]]. The sketch associated with φ for an input sequence *S* is the set of the offsets of all selected *k*-mers from every context of *S*: $M_{\varphi }(S,c)=\underset{i}{\cup }\left\{i+o-1\;|\;\;o\in \varphi (C(S,c,i))\right\}$.

This general sketching definition can model methods with context [where *c *>* *1, e.g. minimizers (Roberts et al. 2004a,b) and minmers (Kille et al. 2023)], and context-free methods [where *c *=* *1, e.g. syncmers (Edgar 2021, Dutta et al. 2022), fractional minimizers (Rouzé et al. 2023)]. We focus on context-free methods, and the selection function makes a binary decision: Do or do not select the one *k*-mer in the context. Such sketching selection function is defined by the set of all *k*-mers that it selects among all possible *σ^k^ k*-mers: $P_{\varphi }=\{m\in \Sigma^{k}\;|\;\varphi (m)\neq \emptyset \}$. Equivalently, the function φ is the indicator function of the set $P_{\varphi }$. Thus, computing the sketch reduces to computing the intersection between the *k*-mers in the sequence and the set $P_{\varphi }$:

$$M_{\varphi }(S,1)=\{i\in [0,|S|-k+1]\;|\;S[i:k]\in P_{\varphi }\}.$$

Going forward, we do not differentiate between the selection function φ and the set $P_{\varphi }$, and we freely use φ with set notations.

### 2.2 Cycles of the de Bruijn graph and selection deserts

A scheme φ has a *strong window guarantee* of *w* if the maximum distance between any two consecutively selected *k*-mers in any sequence is at most *w*. A scheme has a *relaxed window guarantee* if there exist only a small number of well-characterized repetitive sequences not intersecting φ. For example, the low-entropy sequences {AA…,CC…,GG…,TT…} may be used as a set of non-intersecting sequences. When neither holds, a scheme has no window guarantee.

These notions have graph theoretical equivalents using the de Bruijn graph. The de Bruijn graph $B_{k}$ is the directed graph with *σ^k^* nodes (one for each distinct *k*-mer) and an edge u→v when the (k−1)-suffix of *u* is equal to the (k−1)-prefix of *v*. There is a one-to-one correspondence between the sequences of $\Sigma^{*}$ and the walks in $B_{k}$, and a cycle in the de Bruijn graph corresponds to an infinitely long repetitive sequence.

An equivalent definition of the strong window guarantee of *w* is that the graph $B_{k}\setminus \varphi$ (the de Bruijn graph with the *k*-mers of $P_{\varphi }$ removed) is a directed acyclic graph (DAG) and the longest path in this DAG is of length ≤w. In that case, φ is called a *decycling set* of the de Bruijn graph. When $B_{k}\setminus \varphi$ has strongly connected components (SCCs; i.e. it contains cycles), φ does not have a window guarantee for any *w*. Similarly, φ has a relaxed window guarantee if the SCCs contain only cycles of sequences that we are willing to ignore (e.g. the low-entropy sequences shown previously). For a function φ, SCC(φ) denotes the union of all the SCCs of $B_{k}\setminus \varphi$.

Given a *k*-mer $m=m_{0}m_{1}\ldots m_{k-1}$, its rotation *k*-mer is $R(m)=m_{1}\ldots m_{k-1}m_{0}$, and there is an edge m→R(m) in the de Bruijn graph. The cycle of all the rotations of *m* (m,R(m),R(R(m)),…) is called, for historical reasons, a Pure Cycling Register (PCR). The PCRs partition the nodes of the de Bruijn graph and play a special role in describing decycling sets. In particular, a decycling set must contain one node from each PCR and the minimum size decycling sets (MDSs) contain exactly one node from each PCR. Note that the converse is not true: not all sets containing exactly one node from each PCR are decycling. The Mykkeltveit (Mykkeltveit 1972) and Champarnaud (Champarnaud et al. 2004) sets are two examples of MDS constructions.

### 2.3 Sketching methods

The following sketching methods are commonly used by bioinformatics software packages and satisfy the sketching model described above.

The original *window-based* sketching methods are the minimizers (Roberts et al. 2004a,b). The input to the minimizers function is a context of *w k*-mers (originally called a window), and the function returns the index of the lexicographically smallest *k*-mer among the input, which is defined by some pre-determined order on the *k*-mers. Various refinements of minimizers exist such as local schemes (Schleimer et al. 2003) and minmers (Kille et al. 2023). These methods share the important property that they have a context or window length > 1, and they always select at least one *k*-mer in each input window. Consequently, these methods have a strong window guarantee of length equal to the input window length. In some applications, post-processing is applied to not select two identical homopolymer *k*-mers (say AA…A) in a row, to avoid selecting too many *k*-mers in low-entropy regions of the genome. This effectively gives a relaxed window guarantee.

*Positional minimums* methods such as syncmers (Edgar 2021) have a context of length 1. They have extra parameters *s* and *t*, where a *k*-mer *m* is selected if the smallest *s*-mer among the k−s+1*s*-mers of *m* starts at position *t*. As before, the smallest *s*-mer is defined by some pre-determined order on *s*-mers. A notable generalization of syncmers is the parameterized syncmers (Dutta et al. 2022) that use a bit mask of locations for the smallest *s*-mer instead of the parameter *t*.

**Figure 1.**
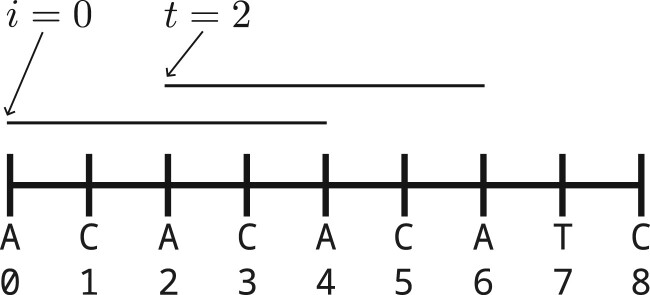
The left-most tie breaking rule implies a relaxed window guarantee for syncmers when t≤s/2. In this example, k=9,t=2,s=5, and the alphabetic order on 5-mers is used. The 9-mer is not selected because the 5-mer at position 2 is equal to the 5-mer at position 0 and the left-most tie breaking rule. Because this 5-mer overlaps with itself, it is “almost” repetitive: It is a sesqui-power $x^{n}y$ with x=AC,y=A,n=2. Repetitive sequences of length *t *=* *2 are skipped over.

When *s*-mers are tied, these methods use a left-most tie breaking rule. This rule has an interesting consequence on the window property. If *t *=* *0 (i.e. the first base), then syncmers have a strong window guarantee, though the length might be as long as *σ^s^* (Marçais et al. 2024). When 0<t≤s/2 and the *s*-mer *m_s_* at position *t* is minimal but not selected because an identical *s*-mer starts at position 0≤i<t, then *m_s_* must be a “sesqui-power,” i.e. a word of the form $m_{s}=x^{n}y$ where *x* is of length *t−i* and *y* is a prefix of *x* (see Fig. 1). Effectively this skips low-entropy genome regions that are repetitive with repeat lengths at most *t*, giving a relaxed window guarantee. When t≥s, there is no longer a window guarantee. Similarly, parameterized syncmers have a strong window guarantee if the bit 0 is set and either a relaxed guarantee or no guarantee for any other value of the mask.

*Hash-based* sketching methods are context-free functions that select a random subset of *k*-mers using a hash function. Examples include when the hash value is equal to 0 mod p or when it is less than or equal to *f* for a pre-determined modulus *p* or fraction *f*. In general, these sketching methods do not have any window guarantee.

## 3 Decycling in *k*-nonical space

In this section, unless specified otherwise, we assume that the sketching function φ is context-free.

### 3.1 Symmetric selection function

Compared to classic string algorithms, bioinformatics algorithms must take into account the double-stranded nature of DNA. In particular, when DNA is sequenced it is usually unknown which strands of the DNA are actually sequenced. In other words, the sequence of a read *r* and its reverse complement sequence ${\bar{r}}^{r}$ carry the same information. Consequently, in algorithms using sketching, it is required to have “symmetric” selection functions, where the same decision is applied to a *k*-mer and its reverse complement. Formally, a selection function is defined to be symmetric when $\varphi (m)=\varphi ({\bar{m}}^{r})$ for every *k*-mer *m*.

Sketching schemes are designed as classical string algorithms, ignoring the required symmetry. Then, in practice, software programs use canonical *k*-mers rather than *k*-mers. Effectively, instead of using the selection function φ they use the *canonicalized function* $\varphi^{c}$ defined by $\varphi^{c}(m)=\varphi (m^{c})$. $\varphi^{c}$ is guaranteed to be symmetric.

It is often assumed that the canonicalized function $\varphi^{c}$ essentially has the same properties as φ, and if anything, $\varphi^{c}$ would select more *k*-mers than φ. In particular, a commonly held belief is that, if the selection function φ has a (relaxed) window guarantee, then so too does $\varphi^{c}$. Unfortunately this does not always hold, especially for the usual context-free methods. Even if a selection function φ has a strong window guarantee, the corresponding $\varphi^{c}$ may not have any window guarantee.

### 3.2 Canonicalized decycling set

In the context-free case, a selection function is equivalent to a set of *k*-mers, and the canonicalized function is obtained via a simple set operation. Using this point of view, we clearly show in the next two Lemmas why $\varphi^{c}$ may not have the same decycling property as φ.

Lemma 1 (Canonicalized function). *The canonicalized selection function* $\varphi^{c}$*of* φ*is defined by the following set:*

$$\varphi^{c}=(\varphi \cap C)\cup ({\bar{\varphi \cap C}}^{r}).$$

*Proof.* By definition of $\varphi^{c}$, for any *k*-mer *m*, $m\in \varphi^{c}\Leftrightarrow m^{c}\in \varphi$. We consider two cases:

- When m∈C: $m\in \varphi^{c}\Leftrightarrow m\in \varphi$. That is, $\varphi^{c}\cap C=\varphi \cap C$.
- when $m\in {\bar{C}}^{r}$: $m\in \varphi^{c}\Leftrightarrow {\bar{m}}^{r}\in \varphi \Leftrightarrow m\in {\bar{\varphi }}^{r}$. That is, $\varphi^{c}\cap {\bar{C}}^{r}={\bar{\varphi }}^{r}\cap {\bar{C}}^{r}={\bar{\varphi \cap C}}^{r}$.

Because $C\cup {\bar{C}}^{r}=\Sigma^{k}$ covers all the *k*-mers, the union of these two cases gives the desired result. □

Lemma 2. *Let* $x_{1}\to x_{2}\to \ldots \to x_{n}\to x_{1}$*be a PCR. Then the reverse complemented k-mers form the PCR* ${\bar{x_{1}}}^{r}\to {\bar{x_{n}}}^{r}\to \ldots \to {\bar{x_{2}}}^{r}\to {\bar{x_{1}}}^{r}$*. These two PCRs may not be disjoint, in which case they are the same PCR.*

*Proof*. The second part comes from the fact that PCRs partition the nodes of the de Bruijn graph.

For the first part, it is sufficient to show that for any *k*-mer *m* there is an edge ${\bar{R(m)}}^{r}\to {\bar{m}}^{r}$ where *R*(*m*) is the rotation of *m*. With $m=m_{0}m_{1}\ldots m_{k-1},\;R(m)=m_{1}\ldots m_{k-1}m_{0}$, then ${\bar{R(m)}}^{r}=\bar{m_{0}}\bar{m_{k-1}}\ldots \bar{m_{1}}$ has an edge to $\bar{m_{k-1}}\ldots \bar{m_{1}}\bar{m_{0}}=R({\bar{R(m)}}^{r})={\bar{m}}^{r}$. □

**Figure 2.**
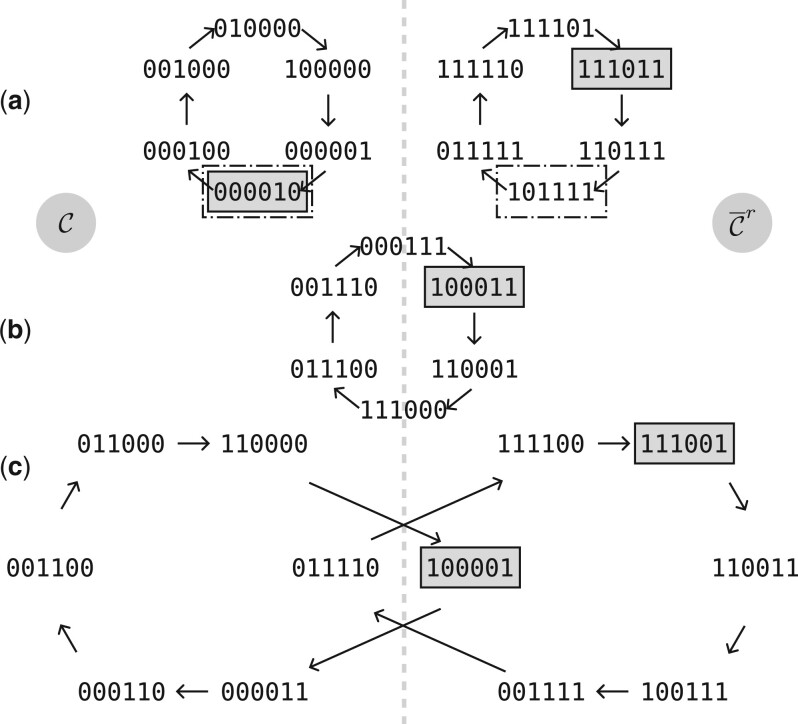
For *k *=* *6 and *σ*  =  2, examples of PCRs. Every *k*-mer on the left side is a canonical *k*-mer (∈C), and *k*-mers symmetrical compared to the vertical line are reverse complement of each other. The *k*-mers inside gray boxes are an example of set φ, and the *k*-mers in dashed boxes are the corresponding $\varphi^{c}$ set. 000010 is in both φ and $\varphi^{c}$ (case a). Not every PCR is covered by $\varphi^{c}$, and consequently $\varphi^{c}$ is not decycling (cases b and c).

Figure 2 shows examples of PCRs for the binary alphabet and *k *=* *6. In this representation, the dashed vertical line acts as a “line of symmetry,” where canonical *k*-mers are on the left and non-canonical *k*-mers are on the right. Cases (a) and (c) are when the PCR and the reverse complement PCR are disjoint. Case (a) is when the PCR is entirely contained in C or ${\bar{C}}^{r}$, i.e. neither the PCR nor the reverse complement cross the dashed line. In case (c), both the PCR and the reverse complement cross the dashed line. Case (b) is when the same PCR contains *k*-mers and their reverse complements. *k*-mers on the dashed line are self-reverse complements.

The set $\varphi^{c}$ is constructed from φ in two steps: (i) only the *k*-mers of φ on the left of the line are selected, and (ii) the symmetrical *k*-mers are added. Consequently, in the cases where the *k*-mers of φ for a PCR and its reverse complement are in ${\bar{C}}^{r}$ (on the right side of the plane), they are not selected in step 1 and $\varphi^{c}$ does not cover these PCRs (see Fig. 2b and c). Hence $\varphi^{c}$ does not necessarily contain a *k*-mer from every PCR, and it is therefore “less decycling” than the original function φ: the SCCs in $\text{SCC}(\varphi^{c})$ are super-sets of SCC(φ) as $\text{SCC}(\varphi^{c})$ also contain, at least, the uncovered PCRs.

Table 1 shows the number of SCCs and their cumulative size as a percent of the total number of *k*-mers (*k *=* *15) for a variety of context-free schemes. The Mykkeltveit and Champarnaud sets are two known construction methods for MDSs. Although these sets are not used on their own as sketching methods, the Mykkeltveit set in particular has been used as a starting point to define sketching methods (Orenstein et al. 2017, 2016, Ekim et al. 2020, Pellow et al. 2023). By construction, these sets are decycling.

**Table 1.** For *σ*  =  4 and *k *=* *15, the table gives the number of SCCs, the percent of *k*-mers of the de Bruijn graph that are in an SCC, and the size of the *k*-mer sets relative to the total number of *k*-mers (4^15^). Syncmers use *s *=* *6 and t∈{0,1,7}. Because Mykkeltveit, Champarnaud, and Syncmers with *t *=* *0 are decycling, there are no SCCs. Syncmers with *t *=* *1 have a relaxed window guarantee that allows the homopolymer sequences, and hence this Syncmer method has only *σ*  =  4 SCCs and 4 nodes in these SCCs. In every case, the canonicalized set leaves large SCCs in $B_{k}\setminus \varphi^{c}$. The relative set size is equal to the expected density (i.e. the percentage of selected *k*-mers in a long random sequence).

	mykkeltveit		champarnaud		fractional		syncmer 0		syncmer 1		syncmer 7	
	φ	$\varphi^{c}$	φ	$\varphi^{c}$	φ	$\varphi^{c}$	φ	$\varphi^{c}$	φ	$\varphi^{c}$	φ	$\varphi^{c}$
Number of SCCs	0	1	0	1	1	1	0	1	4	3	1337	1
SCC cumulative size (%)	0.00	84.35	0.00	95.94	89.98	89.98	0.00	87.23	0.00	84.19	0.01	83.74
Set relative size (%)	6.67	10.73	6.67	4.06	10.00	10.00	10.12	9.95	10.00	10.00	9.99	9.98

The syncmer methods use *s *=* *6 and values of t∈{0,1,7}, along with a random ordering of the *s*-mers. For *t *=* *0 and *t *=* *1, the syncmers have a strong and relaxed window guarantee (four SCCs corresponding to the hompolymer sequences), respectively. For *t *=* *7, because *t *>* s*, there is no window guarantee, and this set leaves many small SCCs. The value t≈k/2 is an often recommended setting for syncmers (Shaw and Yu 2022).

The fractional set selects a fraction of *k*-mers f=1/(k−s+1) (i.e. the fractional set is expected to be of the same size as the syncmer sets, as is observed in Table 1) and uses a random permutation of *k*-mers. This method selects a random subset of all *k*-mers, has no window guarantee, and leaves one very large strongly connected component. It shows that a random set of *k*-mers does not approximate a set with a window guarantee.

After canonicalization, the window guarantees (whether strong or relaxed) do not hold anymore, and the sizes of the SCCs range from 83% to 95% of the de Bruijn graph. The Champarnaud set leads to a much larger component than the Mykkeltveit set, showing that the effect of canonicalization can vary significantly between methods, even with strong window guarantee. Canonicalization does not have a visible effect on the fractional set as it has no guarantee and a large SCC even before canonicalization.

### 3.3 Context-free vs. context-aware methods

Although it is not a requirement of the definition of φ, every sketching method with a context used in practice always selects at least one *k*-mer from their context (e.g. minimizers, minmers). As a consequence, for these sketching methods, the canonicalized function $\varphi^{c}$ defined by $\varphi^{c}(m_{1},\ldots ,m_{n})=\varphi ({\bar{m_{1}}}^{r},\ldots ,{\bar{m_{n}}}^{r})$ also always select at least one *k*-mer from its context. In other words, both φ and $\varphi^{c}$ have, by construction, a strong window guarantee that is equal to the number of *k*-mers in the context.

Many *k*-mer orders have been designed (e.g. Zheng et al. 2020, Hoang et al. 2024, Pissis and Sung 2024) to improve various properties of minimizers. These orders may be only partially defined using a decycling set (aka “compatible orders,” Marçais et al. 2018) such as in (Orenstein et al. 2017, DeBlasio et al. 2019, Ekim et al. 2020, Zheng et al. 2021, Pellow et al. 2023), or implicitly like in the “windowed syncmers” (Dutta et al. 2022). The robustness to canonicalization of minimizers is independent of the chosen order; therefore, every one of these schemes is robust and is consequently not part of the following analysis on sketching deserts.

The situation is very different with context-free methods: Any non-trivial context-free sketching method must not always select a *k*-mer from the context. In fact, there is only one context-free method that always select the one *k*-mer from every context: It is the function that selects every *k*-mer from any input sequence and is equivalent to no sketching at all. Therefore, as seen in the previous section, a context-free method is not guaranteed to be a decycling set before or after canonicalization.

Context-free and context-aware methods with a window (like minimizers) have different trade-offs. Minimizers have a strong window guarantee that extends to *k*-nonical space. On the other hand, because of this window, finding low density minimizers schemes (i.e. minimizers with sparse sketches) is a difficult problem (Marçais et al. 2017, Zheng et al. 2020).

A context-free method is defined by a set and its density can be made arbitrarily small by using a small set or using sub-sampling (Edgar 2021, Rouzé et al. 2023). This comes at the cost of not having any built-in window guarantee. To enforce such a guarantee the set must be carefully chosen (i.e. it must be a decycling set). Finding a set that is also decycling in *k*-nonical space adds another difficulty and new trade-offs. This problem is tackled in Section 5.

### 3.4 Canonicalized sketching deserts

The existence of large SCCs for a given sketching method implies that there exist long—even infinitely long—sequences without any selected *k*-mers. Moreover, the full set of sequences spelled out by these SCCs is usually not known due to the complexity of the components. We call a sufficiently long region of a genome that does not contain any *k*-mers from a sketching method a *sketching deserts*. These regions are either undersampled or skipped by the sketching method. Thus, they are ignored by analyses using these sketching methods.

What precisely constitutes a sketching desert depends on the particular bioinformatics application and the type of data analyzed. Consider an application using *k*-mers from a sketch as anchors for alignment against a genome (Li and Birol 2018, Jain et al. 2022) or using the sketches in an alignment-free method to characterize bacterial strands [e.g. Kraken (Wood and Salzberg 2014)]. In such applications, a minimum number of selected *k*-mers may be needed to (i) pass quality filters and (ii) have significant statistical power. For example, with short-read sequencing (200 or fewer bases) and requiring a minimum of four selected *k*-mers per read, gaps of 50 bases or more between selected *k*-mers are problematic. Thus, regions of the genome of 50 bases or more without selected *k*-mers are considered sketching deserts for these applications.

Table 2 shows the cumulative size of the sketching deserts for three *k*-mer sizes in the human genome (GRCh38) (per-chromosome tables are available in Supplementary Appendix “Chromosome resolution sketching deserts” and full histograms are available as Supplementary data). For the *k *=* *15 case, when switching to *k*-nonical *k*-mers, the size of sketching deserts jumps by two order of magnitude, from a tiny part of the genome to a more sizeable chunk. For example, for syncmer with *t *=* *0 and *k *=* *15 the cumulative desert cumulative size goes from 80 kb to 19.51 Mb. Even though it is still relatively small compared to the genome size (≈0.6%), as is shown below, it is large enough to contain complete exons, and therefore to introduce systematic biases in downstream analysis. The full histograms (provided as Supplementary data) show gaps between selected *k*-mers of up to 1000 bases in *k*-nonical space, compared to at most 70 bases otherwise. The potential size of sketching deserts increases with the length of the *k*-mers, as seen with *k *=* *31, 63 compared to *k *=* *15.

**Table 2.** Cumulative size in mega-bases of the sketching desert of length ≥50,≥75 and ≥150, respectively, for *k *=* *15, 31, 63 in the human genome GRCh38. For methods using a random order (fractional, syncmers), the values are averages over three independent runs. The syncmer method parameters are s=⌊k/2⌋ and t∈{0,1,⌊k/2⌋}. The fractional parameter *f* is set to match the number of selected *k*-mers of the syncmer methods (i.e. f=1/(k−s+1)). In every method, except fractional, the canonicalized set has sketching desert orders of magnitude larger than the original method.

	Mykkeltveit		Champarnaud		fractional		syncmer 0		syncmer 1		**syncmer** k/2	
	φ	$\varphi^{c}$	φ	$\varphi^{c}$	φ	$\varphi^{c}$	φ	$\varphi^{c}$	φ	$\varphi^{c}$	φ	$\varphi^{c}$
k=15,≥50	0.28	15.97	0.81	1009.22	60.45	61.36	0.08	19.51	0.04	13.23	1.73	14.60
k=31,≥75	26.13	139.54	37.33	1320.33	176.76	176.75	3.29	81.15	1.55	70.67	1.95	71.70
k=63,≥150	36.22	241.06	41.10	1252.49	165.66	165.38	2.79	76.57	1.90	70.23	2.14	72.69

Similar effects are observed on the sequence of protein-coding genes. For example, with syncmers (k=15,s=6,t=0), one random order on *s*-mers leaves >20% of the sequence of MIER2 (transcript MIER2-201 with 7084 bases) in sketching deserts, with multiple gaps of > 100 bases, when using canonicalization. Without canonicalization and the same order, there is no gap of 50 bases or more, hence no sketching deserts. Even more pronounced effects are observed with longer *k*-mers: Gene RBBP9 (transcript RBBP9-203 with 1121 bases) has >90% of its sequence in sketching deserts for syncmers (k=31,s=10,t=1) in *k*-nonical space while having no desert before canonicalization.

This also illustrates the impact of the random choice of the order on *s*-mers. For the same syncmers parameters, while for all tested orders there exist transcripts with sketching deserts of >20% of their length, the MIER2 gene has sketching deserts for only one of the tested orders.

### 3.5 SCCs and desert sizes

The relationship between the size of the SCCs with $\varphi^{c}$ (>80% of the *k*-mers) and the length of the sketching deserts (<1% of the sequence) may seem surprising. Two opposite phenomena are at play here.

First, the de Bruijn graph has a high connectivity (*σ*, except for the homopolymers), and a small diameter (*k*). It also has a very large number of cycles (e.g. there are ${\left(\sigma !\right)}^{\sigma^{k-1}}/\sigma^{k}$ Hamiltonian cycles) (Maurer 1992). Intuitively, a slight change to a decycling set can create large SCCs.

Second, for a context-free method, the relative size of its *k*-mer set is equal to the expected density. As is seen in Table 1 for the syncmer methods, the canonicalized set $\varphi^{c}$ has approximately the same relative size as φ. Hence, the expected density is the same in both cases, and therefore the expected distance between selected *k*-mers (which is equal to the inverse of the density) also remains the same.

Despite having the same density, the lack of window guarantee with the canonicalized set implies that long sequences not intersecting $\varphi^{c}$ exist, and these sequences create sketching deserts. See the histograms of distances between selected *k*-mers in Supplementary data having a long tail for the canonicalized set $\varphi^{c}$.

The Mykkeltveit and Champarnaud sets are the only two MDSs for which we have an explicit construction algorithm. There exists a very large collection of MDSs (Marçais et al. 2024) for any parameter *k*. Although the Champarnaud set probably has not been used in practice, the different behavior between the Mykkeltveit and Champarnaud sets shows the dramatic effect canonicalization can have, and the difficulty to predict the canonicalization effect for a given decycling set and target sequence combination.

The effect on the syncmer methods for these random choices of orders on the *s*-mers is less dramatic, although still quite large, especially for large *k*-mers. On the other hand, the relationship between the order used on *s*-mers and the size of the sketching desert is completely unknown and nothing in the method a priori prevents similarly large effects when using an order on *s*-mers that interacts poorly with the human genome.

The fractional set method has the weakest guarantee, with significant sketching deserts before and after canonicalization.

## 4 Canonicalizing sketching methods

In the formula of Lemma 1, the intersection with the set of canonical *k*-mers C is the reason why $\varphi^{c}$ intersects with fewer cycles of the de Bruijn graph, creating larger SCCs and larger sketching deserts. By avoiding this initial intersection, the following simple method creates a symmetric selection function from a given selection function φ:

Definition 1 (Union function). As a set, the “union” selection function is defined by: $\varphi^{u}≜\varphi \cup {\bar{\varphi }}^{r}$.

That is a *k*-mer *m* is selected by $\varphi^{u}$ if either *m* or its reverse complement ${\bar{m}}^{r}$ is selected by φ. Because $\varphi^{u}$ is a super-set of φ, using $\varphi^{u}$ does not introduce any new SCCs or sketching deserts. This scheme can be implemented by using the efficient encoding of (Wittler 2023) where a *k*-mer and its reverse complement are indistinguishable.

This union function is not entirely new. Although motivated by a different goal, it is suggested in Pellow et al. (2023) to use an order for minimizers (the “double decycling set order”) which is based on the set $\varphi^{u}$, where φ is the Mykkeltveit set.

On the other hand, the union selection function is not traditionally used with sketching methods, because the size of the set $\varphi^{u}$ is likely about double that of φ, which could change the behavior of the sketching method. For example, the number of selected *k*-mers in a sequence *S*—approximately $\left|\varphi^{u}\cap K(S)\right|$—is likely to double as well, reducing the sparsity of sketches created by the union sketching function compared to that of the original function [i.e. it affects the *density* (Marçais et al. 2017, 2018) of the sketching method]. By comparison, the canonicalized function, thanks to the intersection with C which contains slightly over half of all the *k*-mers, is expected to have a size similar to that of the original sketching function.

We therefore propose the following optimization problem of finding a function which is as close as possible to the canonicalization function while not increasing the number of SCCs.

Problem 1 (Sparse canonicalization). Given a set φ, find the smallest set *A* such that $\varphi^{c}\subset A$ and SCC(A)⊂SCC(φ).

The union set of Definition 1 satisfies the two conditions of Problem 1, but it is not necessarily of smallest size. We use the following greedy procedure to reduce its size: Starting from a union set $\varphi^{u}$, each *k*-mer is examined one by one, in a random order. If a *k*-mer and its reverse complement can be removed from the set without creating a strongly connected component, they both are removed from the set. Otherwise, the set remains unchanged.

This randomized heuristic is not guaranteed to return the optimal solution. Because the strongly connected component are computed to test whether a *k*-mer can be removed, the runtime is exponential in *k*.

Performance of this procedure is shown in Section 6.

## 5 Symmetric sketching function design

The previous sections focused on the effect of using canonical *k*-mers with existing sketching methods and how to adapt these methods. An alternative approach is to directly design context-free sketching methods in *k*-nonical space, i.e. finding selection functions φ that satisfy $\varphi (m)=\varphi ({\bar{m}}^{r})$ by design. In this section, we develop an efficient ILP procedure that finds a set of *k*-mers of smallest size under the constraints that it satisfies the symmetry condition and has a strong window guarantee of the desired length (i.e. it is decycling, and the longest path in the DAG $B_{k}\setminus \varphi$ is less than some specified parameters).

To do this, we recall preliminary definitions related to feedback vertex sets (FVSs) and nilpotent matrices in the next section. We then define the *Maximum Nilpotent Submatrix Problem* (MNSP) and show that this problem is NP-hard through a reduction from a problem related to decycling. The connection used by this reduction is an algebraic condition on adjacency matrices that leads to an ILP formulation for the MNSP (the proof of correctness for this formulation is in Supplementary Appendix “Circuit Construction”). We specialize this ILP to find symmetric sketching functions by changing the basis on the adjacency matrix used in the construction, which allows us to find a symmetric function that minimizes in order (i) cardinality, (ii) maximum remaining path length, and (iii) expected remaining path length. We conclude with a test of our algorithm on a binary alphabet with *k* ranging from 5 to 8.

### 5.1 FVSs and nilpotency

A FVS of a digraph *G* is a set of vertices W⊆V such that the digraph G∖W induced by the deletion of *W* is a directed acyclic graph. The Minimum Feedback Vertex Set (MFVS) problem is to find the minimum cardinality FVS and was one of the original 21 problems shown to be NP-hard (Karp et al. 1972).

Every MDS solves the MFVS problem on the de Bruijn graph, yet unlike the general problem, there are efficient algorithms to find MDSs (i.e. the Mykkeltveit (1972) and Champarnaud et al. (2004) algorithms). However, the space of MDSs is empirically observed to offer substantial diversity over optimization metrics like the maximum remaining path length, with efficiently selected sets falling short of global optima (Marçais et al. 2024). Moreover, there are no known algorithms to create symmetric MDSs, i.e. sets *M* such that $m\in M\Leftrightarrow {\bar{m}}^{r}\in M$.

To optimize MDSs over both the standard and canonical de Bruijn graph, we develop an ILP formulation for a variant of the MFVS problem that (a) accepts a maximum remaining path length constraint, (b) works with symmetries such as the reverse complement, and (c) minimizes the expected remaining path length after decycling.

Our formulation uses a connection between DAGs and nilpotent matrices to enforce a maximum path length and compute the expected path length. A matrix $A\in R^{n\times n}$ is *nilpotent* when $A^{t}=0$ for some t∈N. The minimal *t* is called the *nilpotent index* of the matrix and is always less than or equal to *n* when it exists (Axler 2015). When *A* is nonnegative (i.e. it has no negative entries), there is a convenient equivalent condition to nilpotence defined with the vector *e* of all 1’s.

Lemma 3. *A nonnegative matrix A is nilpotent if and only if* $A^{t}e=0$*for some* t≤n*. The smallest such t is the nilpotent index of A.*

Proof. If *A* is nilpotent, we have for some t≤n that $A^{t}e=0(e)=0$. If *A* is nonnegative and $A^{t}e=0$, it follows that *A^t^* is the zero matrix because (i) $A^{t}e$ is the vector of row sums, (ii) a nonnegative vector sums to zero if and only if it is the zero vector, and (iii) nonnegative matrices are closed under matrix multiplication. In both directions, *t* can be taken to be minimal with no change, so the smallest such *t* is precisely the nilpotent index. □

Suppose G=(V,E) is a digraph with V={1,2,…,n}. Let $A\in R^{n\times n}$ be the adjacency matrix of *G*, defined as *A_ij_* = 1 if (i,j)∈E and 0 otherwise. A walk in *G* is a string of vertices $v_{0}v_{1}\ldots v_{t}$ such that consecutive vertices are adjacent, i.e. $(v_{i},v_{i+1})\in E$. A path is a walk with no repeated vertices. Recall that $A_{ij}^{t}$ is the number of distinct walks of length *t* between nodes *i* and *j*.

Lemma 4. *G is a DAG if and only if A is nilpotent. Moreover, when G is a DAG, the nilpotent index is one more than the longest path length.*

Proof. Suppose *G* is a DAG. Every walk in *G* is a path because a walk with a repeated vertex would imply the existence of a cycle. As the vertex set is finite, there is a longest path of length $t^{*}<n$. Thus, $A^{t^{*}+1}=0$ since a non-zero entry would indicate a path longer than the longest path. This is the nilpotent index because there are sub-paths of the longest path for every length less than or equal to $t^{*}$.

Now suppose *G* has a cycle. Then *G* also has a walk of every length constructed by starting at any vertex in the cycle and traversing it until reaching the desired length. Thus, $A^{t}\neq 0$ for any *t* because at least one entry must be greater than zero to count the walks around the cycle, showing that *A* is not nilpotent. □

The *maximum t-nilpotent submatrix* (*t*-MNS or MNS) problem is to find a nilpotent submatrix of *A* that maximizes selection with respect to a weight vector *w*, while ensuring the submatrix has a nilpotent index no larger than *t*. Stated differently, the MNS problem is to select a nilpotent submatrix of maximum value, where the value is scored by the sum of fixed weights assigned to each index. The general version of this problem has not been studied, but it is equivalent to MFVS when *A* is an adjacency matrix and *t *=* n*. Moreover, the nonnegative case of *t*-MNS is equivalent to MFVS with an additional max path length constraint for values of *t *<* n*.

Lemma 5. *MNS is NP-hard via a reduction from MFVS.*

Proof. Let *G* be a digraph represented by its adjacency matrix *A*, and let Γ⊆[n] be the indices of a nilpotent submatrix of *A*. By Lemma 4, the induced subgraph $G_{\Gamma }$ is a DAG, so the complement [n]−Γ is a FVS. The same argument taken in reverse shows that a FVS corresponds to a nilpotent submatrix. Thus, every FVS is the complement of an index set for a nilpotent submatrix of *A*. This shows that solutions to MNS for *A* are equivalent to solutions to MFVS for *G* up to a complement. □

### 5.2 MDS-selection ILP

From the proof of Lemma 5, we see that finding a minimum FVS is equivalent to solving *n*-MNS with an adjacency matrix. Moreover, if we constrain the nilpotent index, we constrain the maximum remaining path length of the selected vertices. Thus, we now develop a formulation for the MNS problem over nonnegative matrices that we will use to find path-constrained MDSs. (See Corollary 1 in Supplementary Appendix “ILP Circuit” for an extension of our formulation to the general case.)

For the following, *A* is an *n *×* n* matrix, Γ is an index set, $A_{\Gamma }$ is the submatrix with rows and columns indexed by Γ, $e_{\Gamma }$ is the vector of all 1 s indexed by Γ, and [n] is the set of indices from 1 to *n*. Theorem 1 is proved in Supplementary Appendix “Circuit Construction.”

Theorem 1. *The following formulation solves for a* (t+1)*-nilpotent submatrix, maximizing selection with respect to a weight vector w:*

(1) $$\arg\underset{\Gamma \;\subseteq \;[n]}{\max}\sum_{k\;\in \;\Gamma }w_{k}$$

(2) $$\text{subject}\;\text{to}\;A_{\Gamma }^{t+1}e_{\Gamma }=0$$

Moreover, it can be formulated as a mixed-ILP with O(n) binary variables, O(t·n) continuous variables, and O(t·n) constraints.

When *A* is an adjacency matrix, this formulation solves MFVS with respect to the weights given by *w*. This framework can also model the expected path length of a uniform random walk that stops once a removed vertex is hit.

To do this, we use the random-walk adjacency matrix defined as ${\tilde{A}}_{ij}=A_{ij}/\text{out}(i)$, where out(i) is the number edges originating at vertex *i*. The entry ${\tilde{A}}_{ij}^{t}$ is the probability that a random walk starting at vertex *i* is at vertex *j* during time *t* when uniformly sampling an outgoing edge. For an index set Γ, ${\tilde{A}}_{\Gamma }^{t}$ is also the probability that a random walk starting at vertex *i* is at vertex *j* during time *t*, with the caveat that some states are absorbing, i.e. a random walk stops once a state in [n]−Γ is hit. This leads to two expectation identities given by the random walk.

For random-walk matrix $\tilde{A}$, index set Γ⊆[n], and probability vector $p\in R^{n}$, the expected time before a random walk *X* is absorbed by Γ satisfies

$$E_{p}[X]=e^{⊤}\left(\sum_{k=1}^{\infty }{\tilde{A}}_{\Gamma }^{k}\right)p_{\Gamma },$$

which is equal to infinity when the subset Γ does not asymptotically halt random walks (i.e. it does not represent a decycling set). When Γ induces a submatrix of nilpotent index *t *+* *1 or smaller (i.e. it represents a decycling set), the summation is finite and stops at *k *=* t*.

Thus, using the construction of Supplementary Appendix “ILP Circuit,” we can represent all the vectors used in these expectation formulas. We combine this with Theorem 1 to find a MFVS that secondarily minimizes the expected hitting time of a uniformly random walk using a rescaled uniform probability vector. In Supplementary AppendixFig. A.1, we visualize this process as a probabilistic circuit over the de Bruijn graph with *σ*  =  2 and *k *=* *5.

To work with symmetries, we use a change of basis that considers all equivalent vertices to be the same. For example, if node *v* is equivalent to *w* (e.g. $v={\bar{w}}^{r}$), we simply replace both respective vectors with a new basis vector (v+w)/2 in the adjacency matrix construction.

## 6 Results

We tested our ILP formulation against our greedy union algorithm with the σ=2,4 de Bruijn graph for various values of *k*. For *σ*  =  2, the ILP solved these instances in 2, 10, 30, and 58 minutes, respectively, and required 1 GB of memory. The ILP did not solve the *k *=* *9 instance after 3 hours and peaked at 5 GB of memory, and similarly cannot solve the instances for *σ*  =  4. Because of the exponential growth of the problem size, further optimizations or algorithmic insights are necessary to be able to tackle the larger instances necessary for practical uses. eg, for *σ*  =  4 and *k *=* *15 the de Bruijn graph has over a billion nodes and even smaller values of *k* lead to large ILP formulations with *σ*  =  4 that are out of reach for the current ILP.

Table 3 shows the results of our experiment. The ILP optimized selections have, as expected, a smaller cardinality than the greedy selections. The maximum path lengths of the greedy optimizer are occasionally shorter because the ILP optimizes first for the cardinality of the set.

**Table 3.** Statistics on the solution of the greedy procedure of Section “Canonicalizing sketching methods” and the ILP method. Results are for the de Bruijn graph with σ=2,5≤k≤8 and σ=4,7≤k≤9. The ILP method and the expected path length are only reported for *σ*  =  2 as it is too computationally expensive for *σ*  =  4. The columns show the cardinality of the selected sets, maximum path length after removing the sets from $B_{k}$, and the rounded expected path length after removing the sets. The Mykkeltveit (labeled Mykk.) and Champarnaud (labeled Champ.) sets are used as the starting points of the greedy procedure. The ILP procedure minimizes (in order) for cardinality, maximum path length, and expected path length.

σ	k	Cardinality							Maximum path length							Expected path length		
		Mykk.			Champ.			ILP	Mykk.			Champ.			ILP	Mykk.	Champ.	ILP
		φ	$\varphi^{u}$	$\varphi^{g}$	φ	$\varphi^{u}$	$\varphi^{g}$		φ	$\varphi^{u}$	$\varphi^{g}$	φ	$\varphi^{u}$	$\varphi^{g}$				
2	5	8	14	12	8	14	12	12	11	6	6	11	6	6	6	1.16	1.17	1.36
	6	14	23	19	14	23	19	16	21	15	15	21	15	15	9	1.66	1.72	1.53
	7	20	38	30	20	38	30	28	27	16	16	27	16	16	20	2.12	2.08	1.62
	8	36	69	51	36	65	45	44	39	19	27	47	27	35	23	2.97	2.45	2.0
4	7	2344	4684	3126	2344	4672	2818		111	62	206	141	50	292				
	8	8230	16 440	10 812	8240	16 068	9730		145	89	547	429	109	735				
	9	29 144	28 272	38 546	29 144	58 148	34 538		231	118	1128	520	124	1196				
	10	104 968	209 883	142 681	104 968	207 336	126 502		330	191	2899	1601	289	3317				

Being a symmetric decycling set is a strong condition that is still not well understood theoretically (e.g. the minimum size of a symmetric decycling set is unknown). Similarly to having a window guarantee for sketching methods (see the discussion in Section 3.3), having a window guarantee in *k*-nonical space involves trade-offs (e.g. increased density). Whether the existence of sketching deserts, these deserts actual lengths and locations in the sequence, or the increase in density (or other metrics) is the most important issue is application dependent. These trade-offs should be clearly evaluated when using a sketching method in *k*-nonical space.

## 7 Conclusion

The use of canonical *k*-mers is the standard modification that allows sketching methods for standard text to work with biological sequences. We have shown this has a previously unrecognized flaw when applied to context-free sketching methods. This approach creates sketching deserts that make some sequences effectively invisible to downstream algorithms that use the sketch, potentially creating biases in the analysis. We described the theoretical mechanism behind the creation of these sketching deserts and provided two different options to designing sketching methods that properly handle sequences that are equivalent to their reverse complements. The first method modifies existing sketching methods, but unlike canonical *k*-mers, this modification does not create sketching deserts. The second designs *de novo* symmetric sketching methods that are of minimum size while having small window guarantee.

The proposed methods may not scale to values of *k* large enough for some practical applications. The size of the ILP grows very fast for the DNA alphabet (*σ *= 4) and is unlikely to return an exact solution in a reasonable time. Efficient methods to design symmetrical decycling sets and robust sketching methods is still an open and interesting research avenue.

In general, sketching methods that are used with genomics data must be explicitly designed and validated to handle the equivalence between a sequence and its reverse complement. This must be an intentional step in the design process, not an afterthought.

## Supplementary data

Supplementary data are available at *Oxford Bioinformatics.*

## Supplementary Material

btae629_Supplementary_Data
