# Supplementary figures and images for: k-nonical space: sketching with reverse complements
Source: Bioinformatics. 2024 Oct 21;40(11):btae629. doi: 10.1093/bioinformatics/btae629 (PMC11549021; doi:10.1093/bioinformatics/btae629)

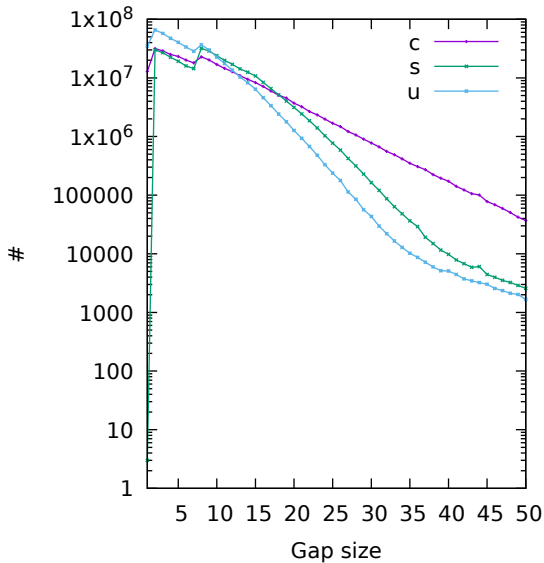

Supplement: btae629_Supplementary_Data [file btae629_supplementary_data.zip › syncmer_7_set_i1.pdf]

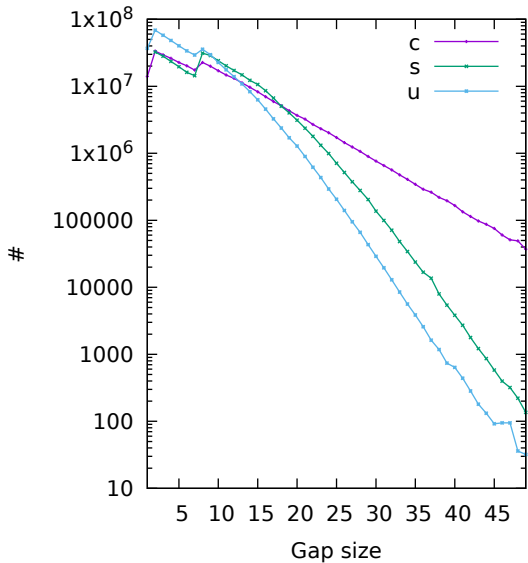

Supplement: btae629_Supplementary_Data [file btae629_supplementary_data.zip › syncmer_1_set_i3.pdf]

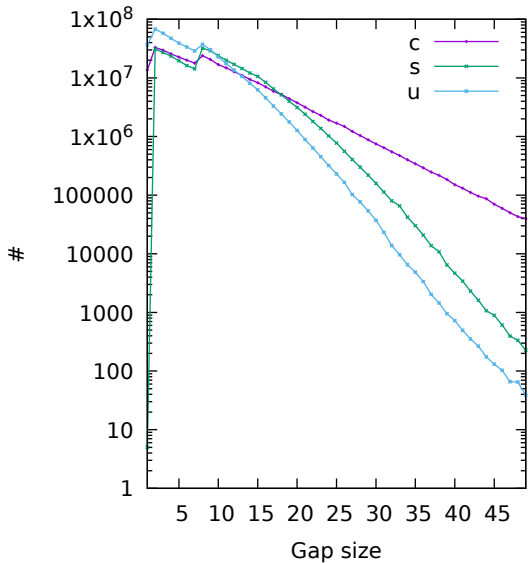

Supplement: btae629_Supplementary_Data [file btae629_supplementary_data.zip › syncmer_1_set_i1.pdf]

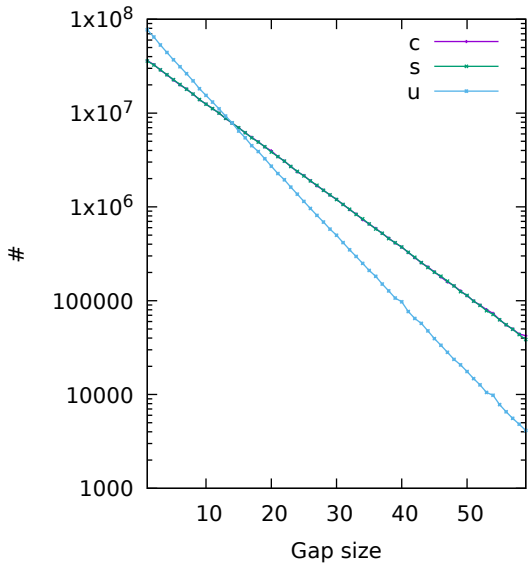

Supplement: btae629_Supplementary_Data [file btae629_supplementary_data.zip › frac_set_i1.pdf]

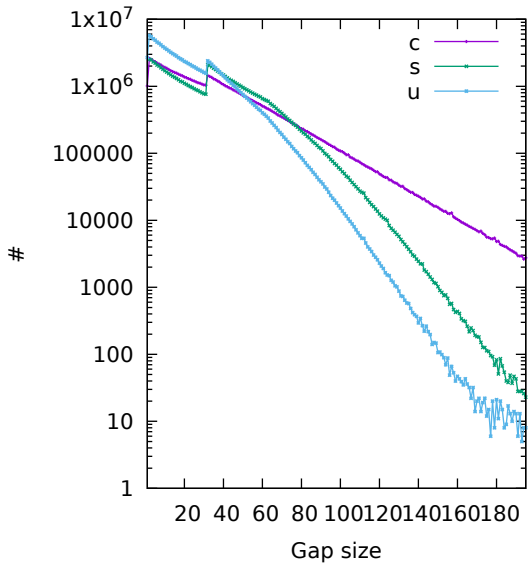

Supplement: btae629_Supplementary_Data [file btae629_supplementary_data.zip › syncmer_31_set_i3.pdf]

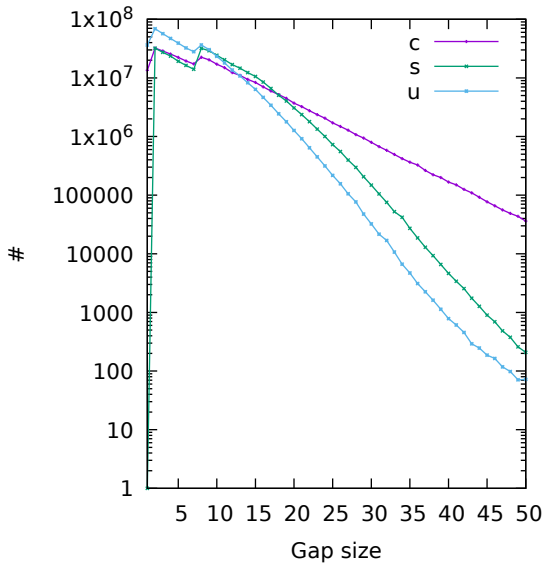

Supplement: btae629_Supplementary_Data [file btae629_supplementary_data.zip › syncmer_1_set_i2.pdf]

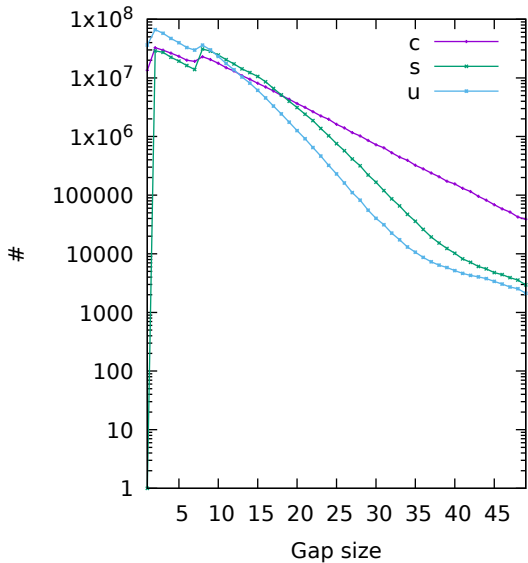

Supplement: btae629_Supplementary_Data [file btae629_supplementary_data.zip › syncmer_7_set_i2.pdf]

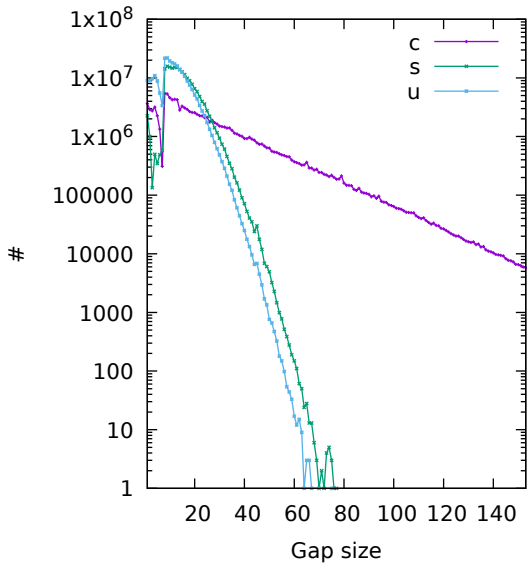

Supplement: btae629_Supplementary_Data [file btae629_supplementary_data.zip › champarnaud_set.pdf]

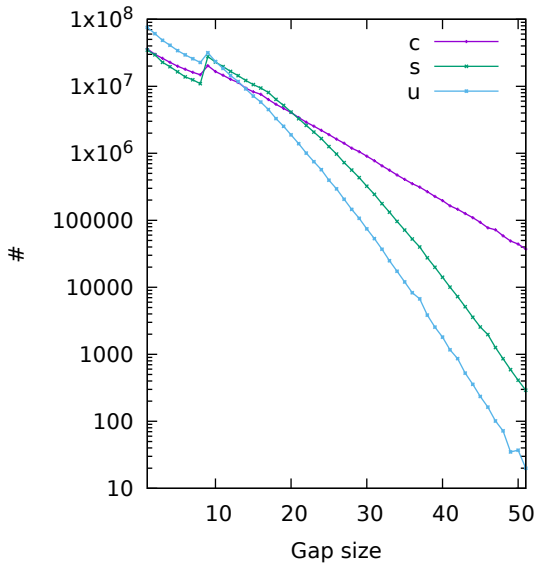

Supplement: btae629_Supplementary_Data [file btae629_supplementary_data.zip › syncmer_0_set_i3.pdf]

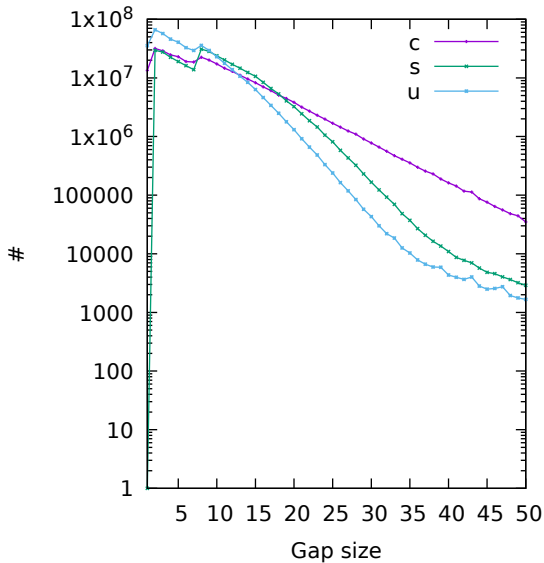

Supplement: btae629_Supplementary_Data [file btae629_supplementary_data.zip › syncmer_7_set_i3.pdf]

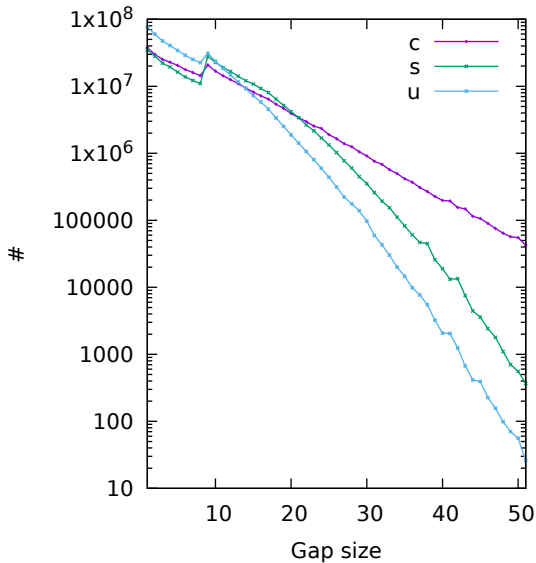

Supplement: btae629_Supplementary_Data [file btae629_supplementary_data.zip › syncmer_0_set_i1.pdf]

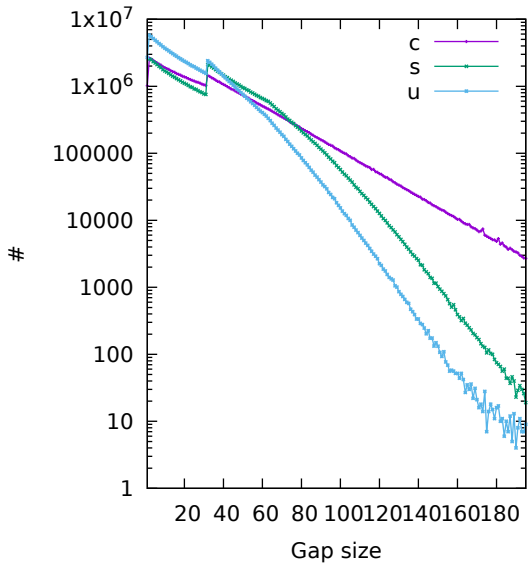

Supplement: btae629_Supplementary_Data [file btae629_supplementary_data.zip › syncmer_31_set_i2.pdf]

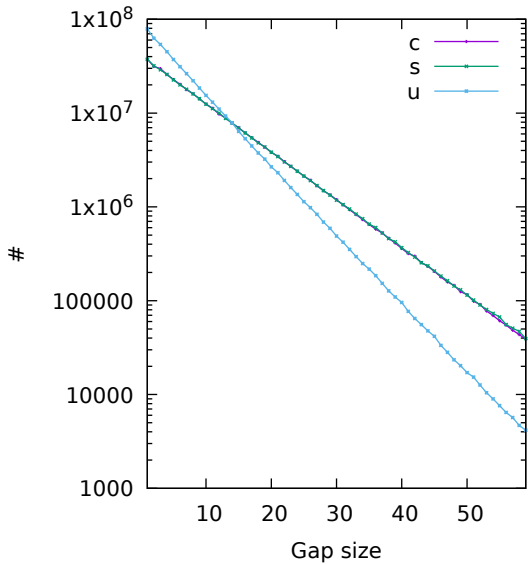

Supplement: btae629_Supplementary_Data [file btae629_supplementary_data.zip › frac_set_i2.pdf]

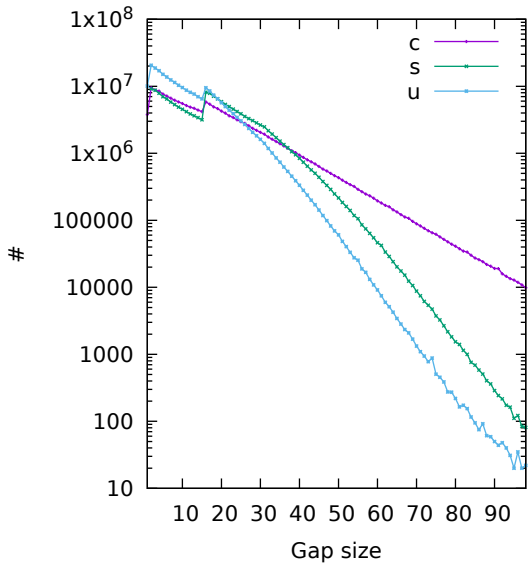

Supplement: btae629_Supplementary_Data [file btae629_supplementary_data.zip › syncmer_15_set_i3.pdf]

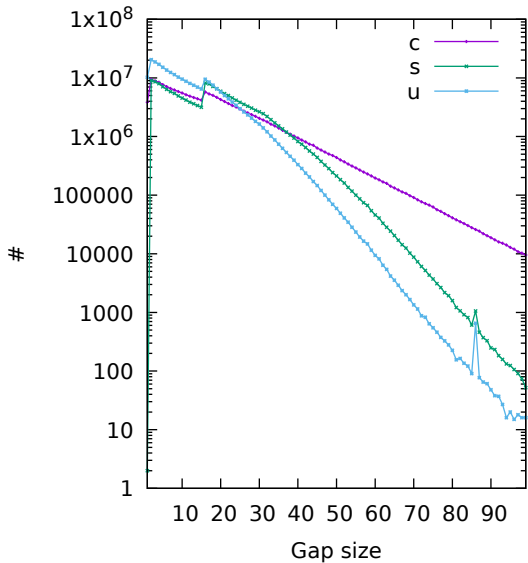

Supplement: btae629_Supplementary_Data [file btae629_supplementary_data.zip › syncmer_15_set_i1.pdf]

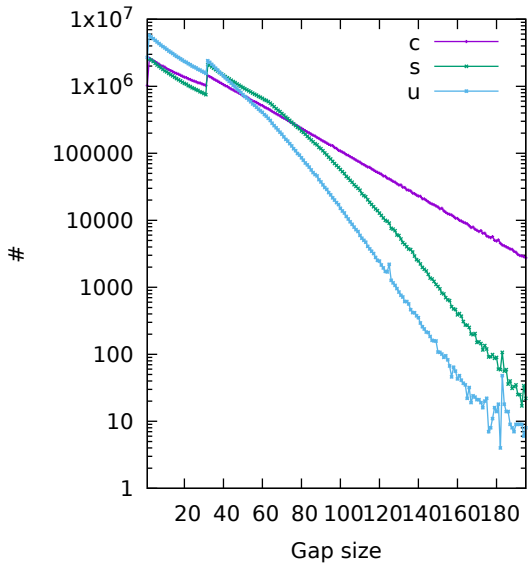

Supplement: btae629_Supplementary_Data [file btae629_supplementary_data.zip › syncmer_31_set_i1.pdf]

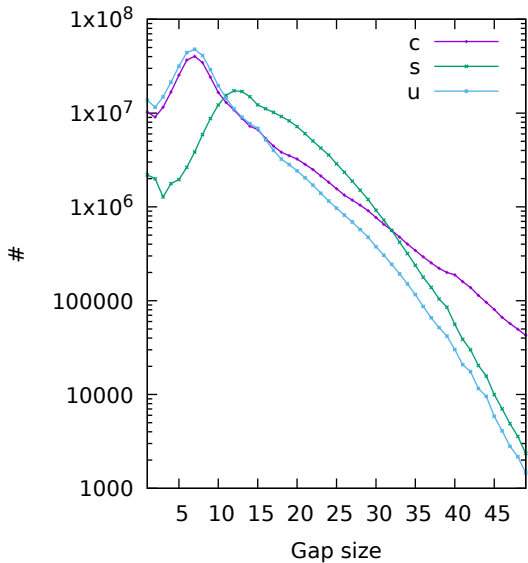

Supplement: btae629_Supplementary_Data [file btae629_supplementary_data.zip › mykkeltveit_set.pdf]

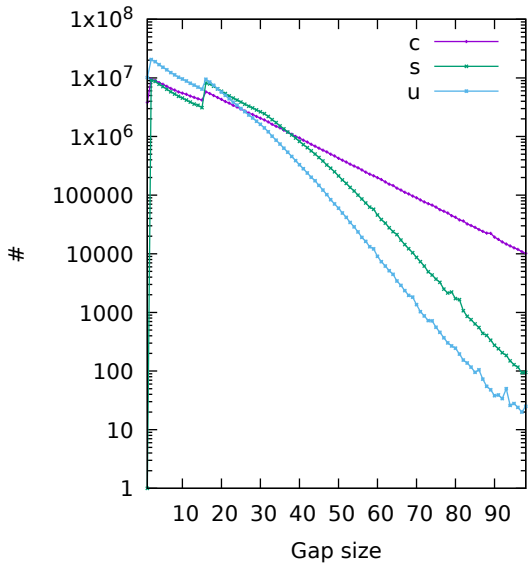

Supplement: btae629_Supplementary_Data [file btae629_supplementary_data.zip › syncmer_15_set_i2.pdf]

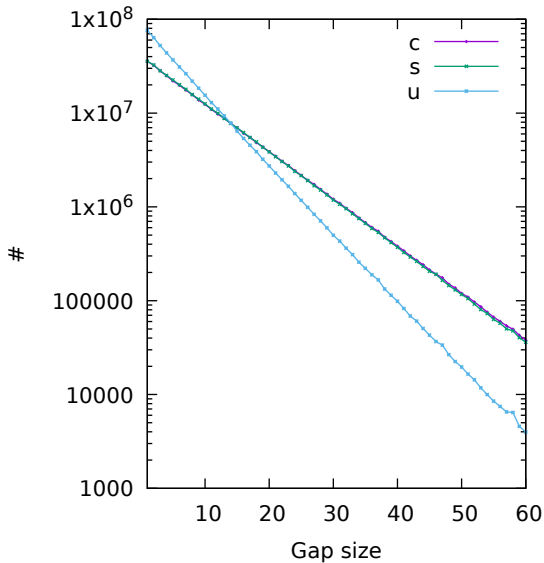

Supplement: btae629_Supplementary_Data [file btae629_supplementary_data.zip › frac_set_i3.pdf]

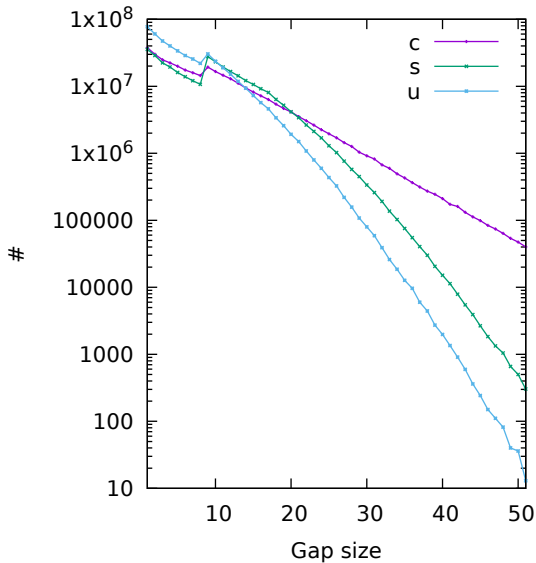

Supplement: btae629_Supplementary_Data [file btae629_supplementary_data.zip › syncmer_0_set_i2.pdf]
