# Supplementary material for: k-nonical space: sketching with reverse complements
Source: Bioinformatics. 2024 Oct 21;40(11):btae629. doi: 10.1093/bioinformatics/btae629 (PMC11549021; doi:10.1093/bioinformatics/btae629)
Supplement: btae629_Supplementary_Data [file btae629_supplementary_data.zip › SupplementalMaterial.zip!/SupplementalMaterial/A4K31/pdfs/syncmer_0_set_i3.pdf]

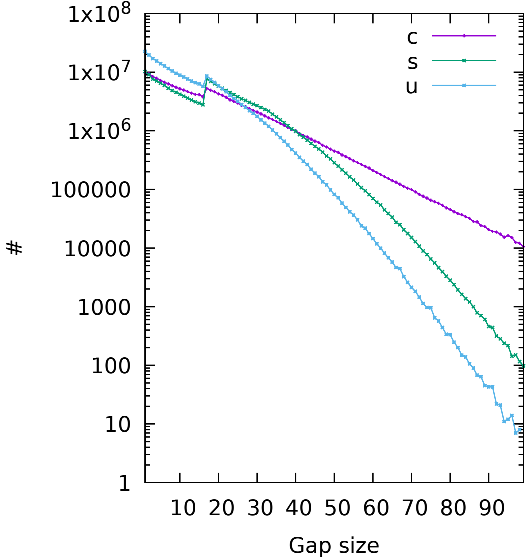

1x10^8
1x10^7
1x10^6
100000
10000
1000
100
10
1
10 20 30 40 50 60 70 80 90
Gap size
#
c
s
u
